# Morphological Transformation in Polymer Composite Materials Filled with Carbon Nanoparticles: Part 1—SEM and XRD Investigations

**DOI:** 10.3390/ma15103531

**Published:** 2022-05-14

**Authors:** Elena Ivan’kova, Igor Kasatkin, Gleb Vaganov, Vladimir Elokhovskiy, Alexander Bugrov, Vladimir Yudin, Ewa Pavlova, Miroslav Slouf

**Affiliations:** 1 Institute of Biosystems and Biotechnology, Peter the Great St. Petersburg Polytechnic University, Polytechnicheskaya ul. 29, 195251 St. Petersburg, Russia; 2 Institute of Macromolecular Compounds, Russian Academy of Sciences, V.O., Bol’shoy pr. 31, 199004 St. Petersburg, Russia; glebvaganov@mail.ru (G.V.); vladimir.elokhovskiy@gmail.com (V.E.); anbugrov@etu.ru (A.B.); yudin@hq.macro.ru (V.Y.); 3 Research Centre for X-ray Diffraction Studies, Saint Petersburg State University, Universitetskaya nab. 7-9, 199034 St. Petersburg, Russia; igor.kasatkin@spbu.ru; 4 Department of Physical Chemistry, Saint Petersburg Electrotechnical University (ETU “LETI”), ul. Professora Popova 5, 197022 St. Petersburg, Russia; 5 Institute of Macromolecular Chemistry, Czech Academy of Sciences, Heyrovského nám. 2, 16206 Prague, Czech Republic; ewapavlova@seznam.cz (E.P.); slouf@imc.cas.cz (M.S.)

**Keywords:** HDPE, carbon nanodiscs, melt-extrusion, fibers, SEM, WAXS, rheology, structure

## Abstract

HDPE-based nanocomposite fibers have been extruded from a melt and drawn up to draw ratio DR = 8. Two kinds of carbon nanodiscs (original ones and those exposed to additional annealing) have been used as fillers. Obtained nanocomposite fibers have been investigated with the help of different experimental methods: rheology, SEM and WAXS. It has been demonstrated that the annealed carbon nanodiscs possess a nucleation ability that finally leads to strong transformation of the material morphology.

## 1. Introduction

High density polyethylene (HDPE) is a semicrystalline thermoplastic polymer that is easily processed due to its not very high melting point, low glass transition temperature and rapid crystallization. The processing of PE into fibers makes it possible to optimize the structure and morphology of the final filament to obtain high mechanical properties [1,2,3,4]. The main factors affecting the different properties of the HDPE fibers include the morphology and spatial distribution of crystalline and amorphous domains along the length and cross section of the filament. For flexible linear polymers such as HDPE, the improvement in mechanical properties comes from creating a bulk flow state that stretches the polymer chains, causing them to form bundles of long semi-crystalline fibrils. The extensional flow required to unfold flexible chains can be achieved by orientational drawing of the fibers obtained by melt spinning at a temperature below the equilibrium melting point of PE [4]. Higgins and Bryant hypothesized that the melt-spun PE fibers have a kind of stress-crystallization morphology [1]. At the same time, the hydrodynamic flow created inside the fibers during extrusion from the melt makes it possible to control the texture of the fiber crystals and hence their final performance characteristics.

Melt-extruded HDPE fibers are of great interest from the point of view of practical use and for the investigation of polymer structures. Usually, such fibers can possess a certain orientation of crystallites (texture), which coincides with the extrusion direction. The melt-extruded PE fibers and films were studied in a large number of works by different experimental methods [5,6,7,8,9,10,11,12,13,14,15,16,17,18,19,20]. It was reported that lamellae with ***a***-texture forms during the extrusion process. At the stage of orientational drawing, a transition from ***a***-texture to highly oriented c-texture occurs. 

Improving the orientation of molecules and increasing the degree of crystallinity of the HDPE fibers by optimizing the process of drawing from the melt are the main tools for enhancing their properties. However, with the advent of nanotechnology, new opportunities have opened up for modifying the deformation-strength characteristics of the polyethylene-based fibers. It is known that nanosized fillers can act as nucleation centers of crystallization, which play a decisive role in regulating the mechanical properties of microfibers. For example, melt spun of HDPE with 1 wt.% cellulose nanofibers was used to obtain the composite fibers with higher values of tensile modulus and strength in comparison with unfilled systems [21,22]. At the same time, structural studies have shown that the increase in the mechanical properties of the fibers is due not to the reinforcing effect of the filler but to an improvement in the orientation of the HDPE molecules in its presence. In another study, organically modified hydrotalcite particles were used as HDPE fiber crystallization nucleants [23]. Carbon nanotubes were also very intensively incorporated into the HDPE matrix, resulting in the formation of a so-called hybrid shish-kebab structure [24,25,26,27,28,29].

Typically, it is assumed that nanoparticles possessing a high aspect ratio (carbon nanotubes and nanofibers, for example) could be the best fillers for the composite polymeric materials since they have high mechanical characteristics (strength at break and Young’s modules) and also can act as nucleating agents, improving the macromolecules orientation along the prepared fibers. We decided to look at this situation from the other side, namely, to find out whether the nanoparticles of a completely different shape are capable of changing the morphology of the composite fibers obtained from the melt and, as a result, their properties. In addition, it is assumed that the presence or absence of the ability of the nanoparticles to act as a nucleating agent should significantly affect the structure and properties of the resulting fibers. 

In the present work, we decided to dope HDPE with rather unusual carbon nanofillers, which have the same shape (nanodiscs) but, as expected, different nucleation abilities. It is supposed that the use of these nanoparticles can lead to drastic changes in the polymer matrix morphology that will subsequently result in altered properties of the melt extruded fibers based on HDPE.

The main aim of this paper was to conduct a detailed morphological investigation of the HDPE-based nanocomposite fibers produced by the melt extrusion process. It is proposed that the results obtained may demonstrate the structural transformation occurring in the samples filled with two kinds of the carbon nanoparticles.

## 2. Materials and Methods

High density polyethylene (HDPE) has been used as the matrix to produce the nanocomposite fibers by melt technology. Two kinds of nanoparticles, i.e., the carbon nanodiscs in the original state (oND) and ones exposed to additional annealing at 2500 °C (aND), have been chosen as fillers. The concentrations of both types of the nanodiscs have been varied, namely, 1, 3, 5 and 10 wt.%.

The dispersing of the filler in the polymer melt as well as the nanocomposite fibers extrusion was carried out by a twin screw microcompounder DSM Xplore (Xplore Instruments, Sittard, The Netherlands) equipped with a special fiber preparation unit (DSM Film Device Machine, Xplore Instruments, Sittard, The Netherlands). The components were mixed for 5 min at 160 °C and a screw rotation speed of 50 rpm. After that, the formation of the nanocomposite fibers was carried out via fixed circular die with subsequent cooling by a flat jet of compressed air of the so-called “air knife” (*p* = 3.9 Pa) just after leaving the spinneret. The cooled fiber has been wound on a receiver coil at a constant speed. Finally, the undrawn fibers containing different concentrations of the two types of the carbon nanodiscs have been obtained.

The prepared fibers have been subjected to high-temperature orientational drawing up to a draw ratio DR = 8 using special home-made equipment. Drawing was carried out in two stages: the fibers were initially drawn up to DR = 4 at 65 °C and then another two times at 100 °C.

The rheological properties of the HDPE melts have been examined by the rheometer Physica MCR301 (Anton Paar, Graz, Austria) and the measuring unit as an in-plane cone CP25-2 (diameter of 25 mm, angle of 2°, the gap between the cone and the plane was 0.05 mm). The test was performed in shear mode at 160 °C, and shear rates varied from 1 to 0.01 s^−1^. The yield point was determined using the empirical Cross equation with the addition of the yield point. The Cross equation with the yield point is the following:

(1) $$\begin{array}{l} \tau (\dot{\gamma })=\tau_{0}+\eta_{\infty }\dot{\gamma }+\frac{(\eta_{0}-\eta_{\infty })\dot{\gamma }}{1\;+\;{\left(\theta \dot{\gamma }\right)}^{p}}\\ \eta (\dot{\gamma })=\frac{\tau_{0}}{\dot{\gamma }}+\eta_{\infty }+(\eta_{0}-\eta_{\infty }){\left[1\;+\;{\left(\theta \dot{\gamma }\right)}^{p}\right]}^{-1} \end{array}$$

where $\tau (\dot{\gamma })$—shear stress (Pa) versus strain rate (s^−1^); $\tau_{0}$—yield point, Pa; $\eta (\dot{\gamma }),\;\eta_{0},\;\eta_{\infty }$—effective viscosity, highest and lowest Newtonian viscosity, Pa·s; *θ*—relaxation time, s; ***p***—exponent is 2/3. After processing the experimental curves of the rheological flow of the studied compositions in accordance with the Cross equation (given above), the yield point was determined.

The structure of the HDPE-based composite fibers has been investigated using the scanning electron microscopes SUPRA 55VP (Carl Zeiss, Oberkochen, Germany) and Quanta 200 FEG (FEI, Hillsboro, OR, USA). The smooth surfaces of the studied samples have been prepared by cryo-ultramicrotomy (sample and knife temperatures −50 and −120 °C, respectively). Then, the smooth cut surfaces have been etched with permanganic mixture (a 1:1 mixture of conc. H_2_SO_4_ and conc. H_3_PO_4_ containing 1 wt.% of KMnO_4_) and washed carefully four times in order to avoid possible etching artifacts [30,31]. After that, the etched specimens have been sputtered with a thin Pt layer, placed into the SEM microscope and observed in secondary electrons (SE) mode at accelerating voltage in the range of 3–10 kV.

The crystalline structure of the HDPE-based nanocomposite fibers has been investigated by 2D-WAXS using the diffractometer Bruker D8 DISCOVER (Bruker, Karlsruhe, Germany) with point focus, 0.5 mm spot size and parallel beam filtered CuKα radiation, as well as an Imagine Plate area detector (Anton Paar, Graz, Austria). Azimuthal intensity distribution profiles have been registered for the main peaks (110) and (200) for all the studied fibers.

## 3. Results

### 3.1. Rheology

**Figure 1:**
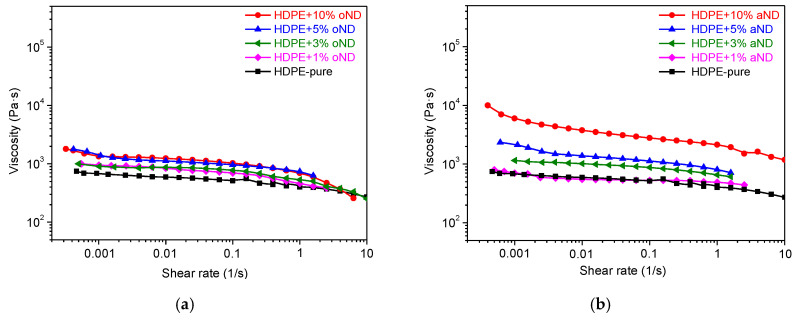
Dependences of the viscosity on the shear rate for the HDPE-based composite melts with the added nanoparticles of oND (**a**) and aND (**b**).

In the first step, the impact of the nanofiller morphology on the rheology of the nanocomposite melts was investigated. For this purpose, the rheological properties of all of the studied compositions (0, 1, 3, 5 and 10 wt.% of the original (oND) and annealed (aND) nanodiscs) based on HDPE were investigated at a constant temperature of 160 °C (i.e., the temperature of the fibers production). The obtained influences of shear rate (*γ*) on the viscosity (*η*) of the polymer melts with the introduced oND (a) and aND (b) nanoparticles are shown in Figure 1.

All of the studied compounds were revealed to be non-Newtonian fluids. The addition of the nanoparticles of various amounts shows different effects on the rheological properties of the composite polymeric melts: (1) the addition of the original nanodiscs does not essentially change the rheological behavior of the melts, which follows from the similarity of the dependencies *η*(*γ*) for the pure HDPE melt and compositions with oND; (2) the presence of the annealed nanodiscs increases melt viscosity, especially at 10 wt.% content. This sharp increase in the viscosity at low shear rates indicates the formation of a physical network structure between the polymer and the nanoparticles and, hence, an appearance of a yield point. Since such structural nets have the bonds of the physical nature, they can break when the shear rate becomes high enough (≥0.01 s^−1^), and, as result, the viscosity of the melt goes down. Thus, on the one hand, if the shear rate is low, the crucial role is played by the structure formed between the nanofiller particles (aND) and the polymer melt; on the other hand, at high shear rates, a dominant role for the mixture with the destroyed structure is mainly determined by the viscosity of the dispersion environment.

**Figure 2:**
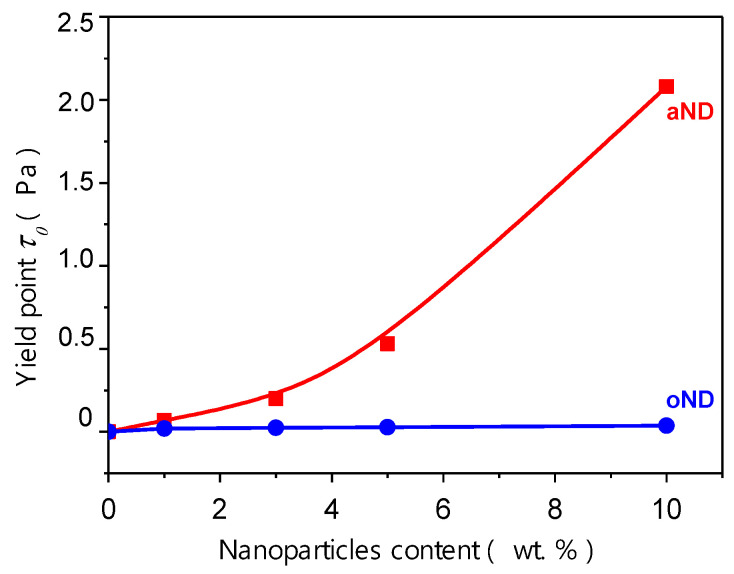
Effect of the nanoparticles content on the yield points of the HDPE-based composite fibers.

As mentioned earlier, the inclusion of the aND into the polyethylene melt leads to the appearance of the yield point—the highest value (2.08 Pa) is found for the melt with 10 wt.% aND (see Figure 2). As for the melt of pure HDPE and all of the compositions with oND, their yield points are too small.

The rheological study of the HDPE-based composite materials at the stage of melt processing detected an evident influence of the introduced nanoparticles on the structuring ability of the polymer melts.

### 3.2. Scanning Electron Microscopy (SEM)

**Figure 3:**
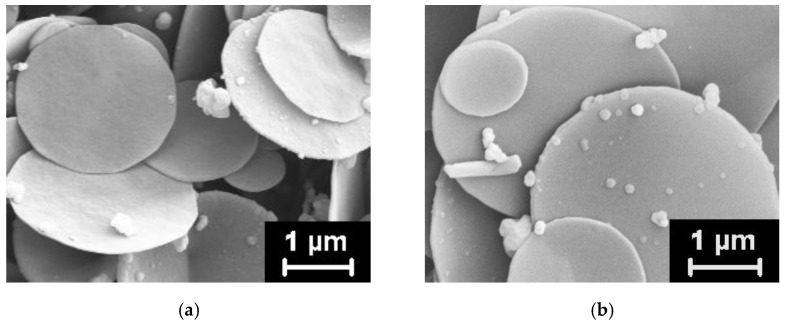
Micrographs of the original (**a**) and high-temperature annealed (**b**) nanodiscs.

The nanodiscs before and after annealing, which were used as fillers in the HDPE-based composite fibers, are shown in Figure 3. Both types of the nanoparticles look identical and have rather big variations in their sizes (0.3–4.8 μm).

**Figure 4:**
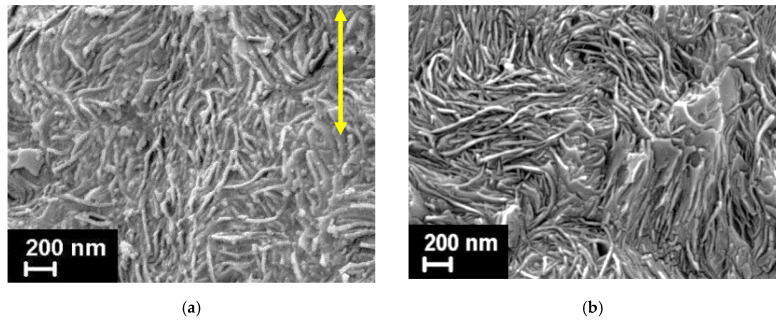
SEM micrographs of the pure HDPE fiber (after etching): (**a**) fiber surface; (**b**) fiber cross-section. An arrow indicates the fiber extrusion direction.

**Figure 5:**
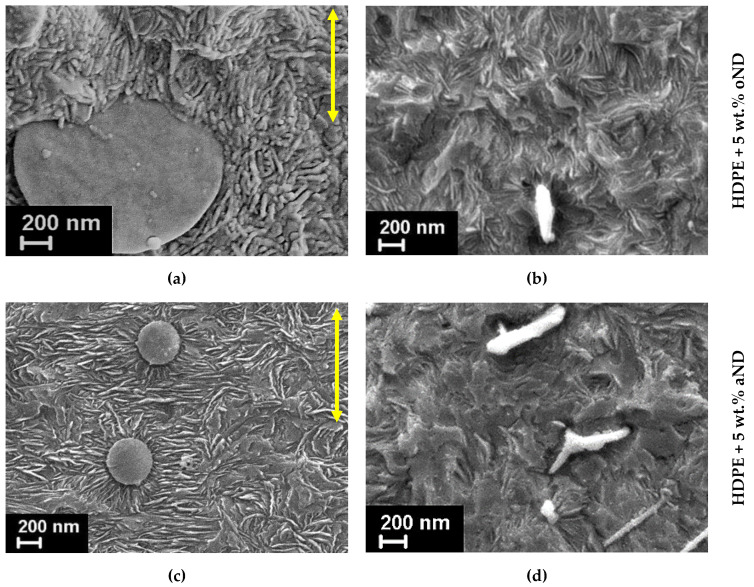
SEM micrographs of the longitudinal (left side—(**a**,**c**)) and transversal (right side—(**b**,**d**)) fracture surfaces of the HDPE-based composite fibers filled with 5 wt.% of the nanodiscs. An arrow indicates the fibers’ extrusion direction.

SEM was employed as well to visualize the crystalline HDPE lamellae and the dispersion of the filler both on the surface and inside the prepared fibers (i.e., on the cryo-cleaved fracture surfaces). The obtained results are shown in Figure 4 and Figure 5.

The morphology of the pure HDPE fiber surface is presented in Figure 4a: the lamellae predominantly oriented along the fiber axis (i.e., parallel to the fiber extrusion direction) can be evidently seen. In Figure 4b, where the cross-section structure is demonstrated, the lamellae edges are found, which also supports the aforementioned observation concerning the lamellae oriented mainly parallel to the fiber axis.

The nanofillers distribution and interaction with the polyethylene matrix was examined on the cryo-cleaved fracture surfaces of the fibers. Figure 5 shows the micrographs of the transverse and longitudinal fracture surfaces of the fibers filled with 5 wt.% of both types of the nanodiscs (oND and aND). The composite fibers doped with other amounts of the nanofillers were also studied with the help of SEM; however, the differences between the sample structures were only in the number of the visible nanodiscs.

In the obtained SEM images, one can see that both types of the nanoparticles (oND and aND) are very well dispersed in the HDPE matrix. It is also worth noting that none of the nanodisc types demonstrate a good adhesion to the polymer matrix, i.e., the nanoparticles pulled out from the HDPE matrix, and their surfaces are found to be free of polymer traces. Surely, this must have an impact on the future behavior and properties of the composite fibers.

The interesting fact that must be noticed is that only one type of the carbon nanodiscs, namely, the annealed ones (aND), possesses the visible nucleation ability. Therefore, on their surface, polyethylene transcrystallites start forming (Figure 5c). These polymeric transcrystallites grow normally to the surface of the nanodiscs introduced, and a sun-like morphology is observed around the nanodiscs. The transcrystallization phenomenon has been revealed and described previously for PE and for other semi-crystalline polymers [25,27,32,33,34]. It turned out that one and the same nanoparticles cannot act as nucleating agents for all of the polymers. It was assumed that one of the most important parameters is the mismatch between the cell parameters of the nanoparticles and the polymer crystallites—if the mismatch does not exceed 15%, the ability to nucleate occurs. The annealed nanodiscs have a very good crystal structure, as will be confirmed below by WAXS. It is assumed that the mismatch between the cell parameters of the carbon nanodiscs and the PE crystallites is low enough to make these nanoparticles capable of nucleation.

Looking at the transversal fracture surfaces of the nanocomposite fibers (Figure 5b,d) one can clearly observe the evident difference in the orientation of the HDPE matrix lamellae. If the fiber is filled by oND, most of the PE lamellae are aligned along the fiber axis, and, therefore, their edges are well seen. At the same time, when the aND nanoparticles are added into the composite fiber, the HDPE lamellae planes are oriented normally to the fiber extrusion direction. Looking ahead, we can state that this very important observation will be essentially supported the WAXS results showing the difference between the ***a***- and ***c***-texture of the polyethylene crystalline structure.

**Figure 6:**
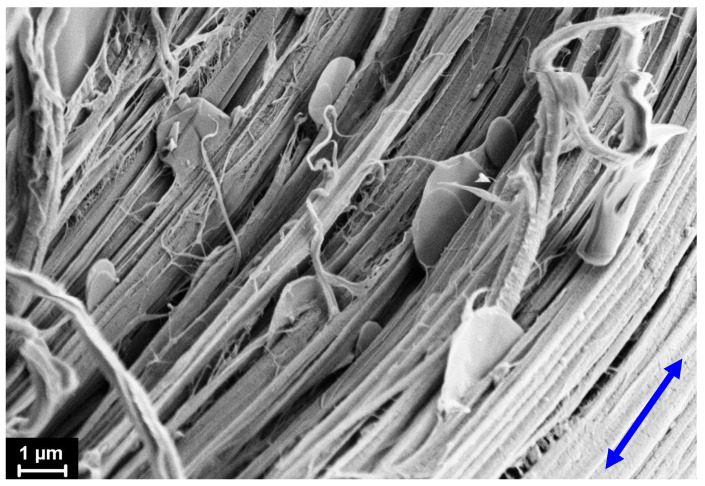
Scanning electron micrograph of the composite fiber drawn up to DR = 8. An arrow indicates the fiber drawing direction.

The morphology of the drawn nanocomposite fibers (DR = 8) was also examined using the SEM method. It is well known [3,32,33,34,35,36,37,38] that during high-temperature orientational drawing, the lamellae morphology transforms in the fibrillar one. As an example, the SEM micrograph of the internal structure of the fiber with the added nanodiscs is presented in Figure 6.

The introduced carbon nanoparticles are found to be oriented along the fiber axis. A remarkable fact is that, since there is very low adhesion between the nanoparticles and the HDPE matrix, these particles do not interfere with the orientation process of the polymer. Nevertheless, we were not able to draw the samples with 10 wt.% of the nanofillers. The main reason for this is assumed to be the low level of adhesion between the nanodiscs and the polyethylene matrix, which allows one to consider these nanoparticles as numerous defects within the polymer. When the concentration of these defects is high enough (10 wt.%), under applied drawing stress, the polymer matrix can be destroyed earlier, and then the transformation into the fibrillar structure occurs, i.e., the destruction processes prevail over the hardening.

### 3.3. WAXS

**Figure 7:**
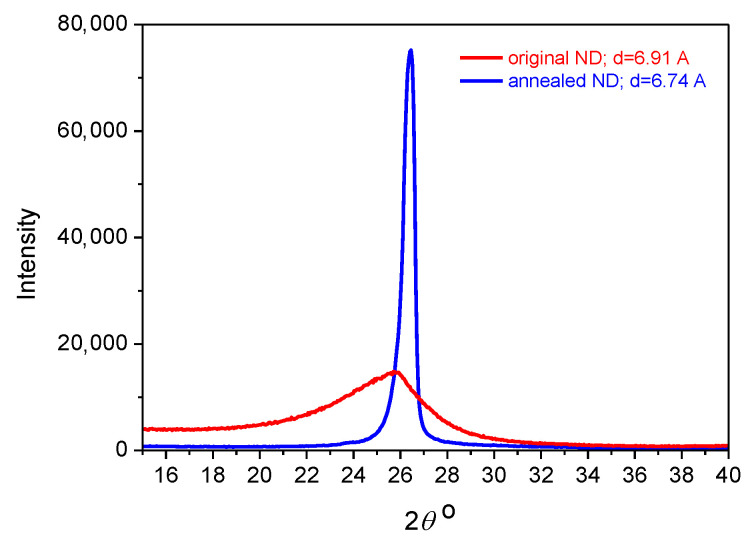
WAXS diffractograms of the original and annealed nanodiscs.

For the first step of the WAXS investigation, a crystal structure of the carbon nanodiscs before and after high-temperature annealing was examined. Figure 7 presents X-ray diffractograms recorded from the nanoparticles of both types. An essential difference on the half-width of the main (002) reflex is clearly revealed, which allows one to draw the following conclusions: the original nanodiscs (oND) have a purely organized inner structure (i.e., they are almost amorphous), while the nanodiscs exposed to high-temperature annealing (aND) possess a crystalline structure with a high level of perfection. It is supposed that both kinds of the nanoparticles will show rather different influences on the morphology and, as a consequence, on the other properties of the composite polymer fibers.

**Figure 8:**
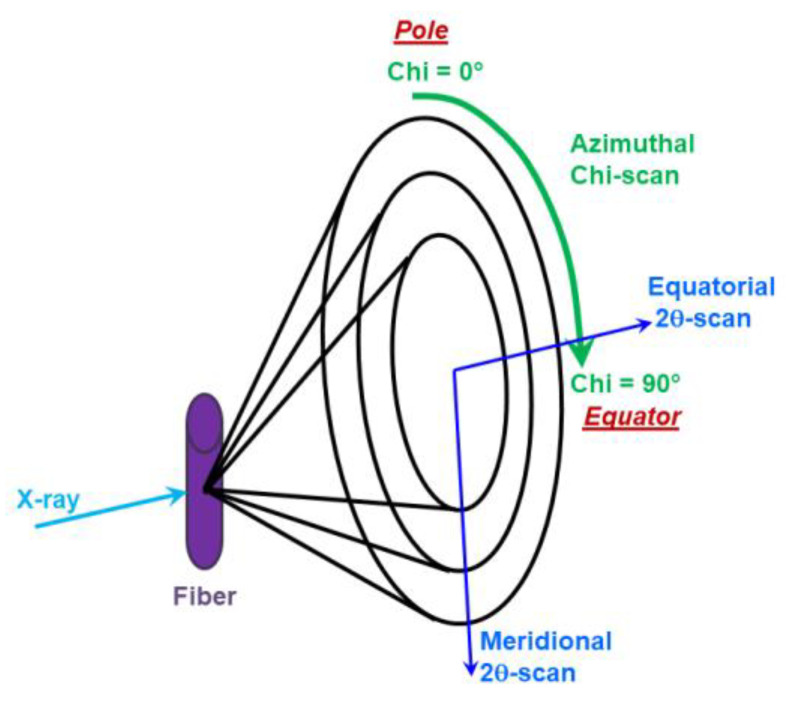
Scheme of the WAXS experiments.

The polyethylene crystalline structure of the studied HDPE-based nanocomposite fibers was also investigated in detail by WAXS. Figure 8 demonstrates a schematic diagram of the WAXS experiments performed for the examination of the HDPE crystalline texture features.

Using 1D-patterns recorded in the *θ*–2*θ* mode along the meridian or equator in the angle range of 2*θ* = 5–80° (see Figure 8), one can extract the most important information concerning a type of crystal structure, as well as the size of coherent scattering regions. To avoid overloading of the paper, we will not give all of the equatorial and meridional WAXS patterns for all types of the samples. Treatment of the thus obtained X-ray data showed that only crystallites of orthorhombic modification were present in all of the investigated samples. It is also worth noting that the average crystallite size in the direction perpendicular to the (110) and (002) planes remained practically unchanged for all of the unoriented fibers (DR = 1) and amounted to D_110_ ≈ 28 nm, D_002_ ≈ 14 nm (the deviation is lower than 10%).

As for the oriented fibers (DR = 8), the most important is the crystallite size along the fiber axis orientation, i.e., along the [002] crystallographic direction. According to our calculations, the average value amounted to D_002_ ≈ 16 nm. Thus, it can be assumed that the introduction of both types of the nanoparticles does not make significant changes in the size of the crystalline regions of the HDPE matrix (the same was concluded elsewhere [39]).

**Figure 9:**
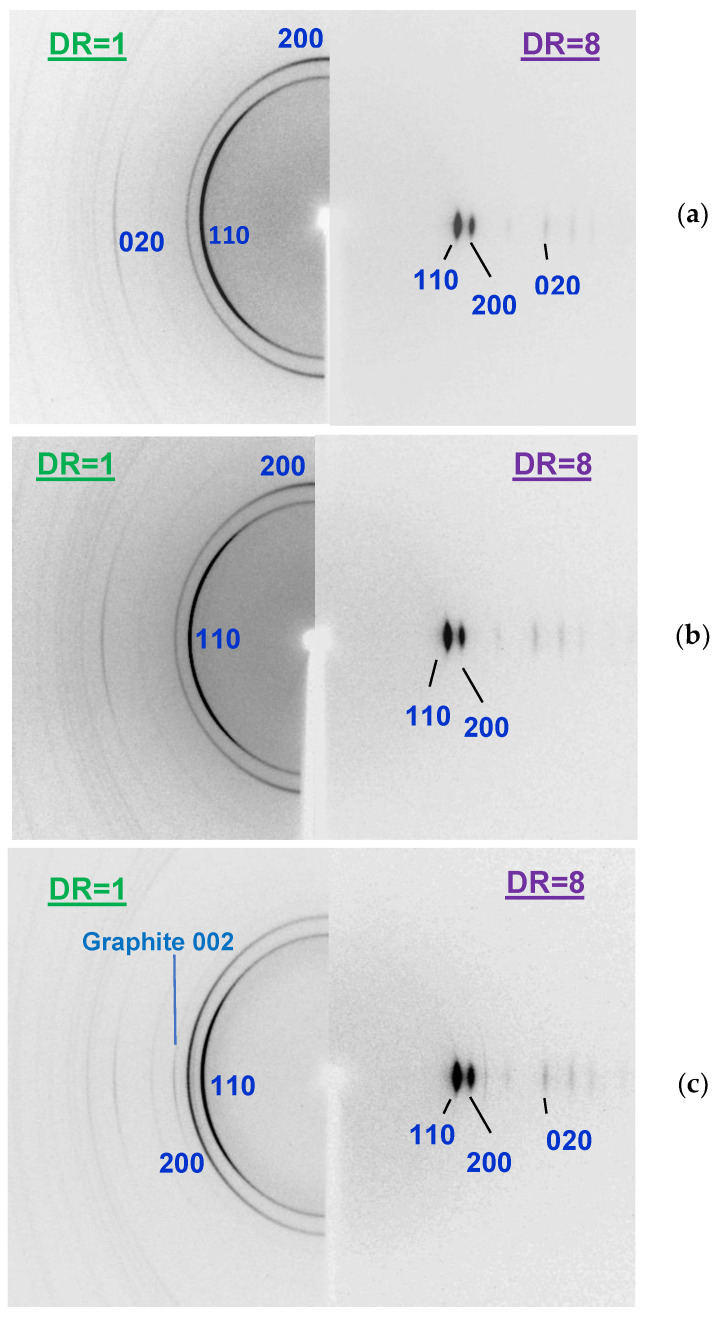
X-ray 2D-patterns of the polyethylene-based fibers before (DR = 1) and after orientational drawing (DR = 8): (**a**) pure HDPE; (**b**) HDPE + 5 wt.% oND; (**c**) HDPE + 5 wt.% aND. The fiber axis was vertical.

In Figure 9, three WAXS 2D-patterns are demonstrated, which are characteristic of the undrawn pure HDPE (Figure 9a) and the composite fibers with the addition of both types of the nanodiscs (Figure 9b,c). It should be noted that, in the samples of the pure HDPE and those doped with oND, the reflex corresponding to the (200) planes is located on the pole—i.e., it is the meridional reflex (Figure 9a,b). For the fibers filled with the annealed nanodiscs (aND) at concentrations of ≥5 wt.%, it is shifted to the equator (see Figure 9c). Also notable is the appearance of the graphite 002 arc-reflex, characterizing the presence of the crystalline aND particles, while the amorphous oND does not demonstrate any X-ray reflex. This indicates that all of the studied unoriented fibers after melt extrusion have some preferred orientation (texture) of the crystalline regions relative to the fiber axis. However, this texture is undergoing significant changes with the incorporation of a large number of the aND nanoparticles into the composite fibers.

Taking into account the results mentioned above, we decided to study the polymer texture features in more detail (i.e., the preferential orientation of the HDPE crystallites according to the fiber axis). As a result, Chi-scans presenting the X-ray intensity distribution along the Debye rings (from the pole to the equator) were recorded according to the scheme in Figure 8.

**Figure 10:**
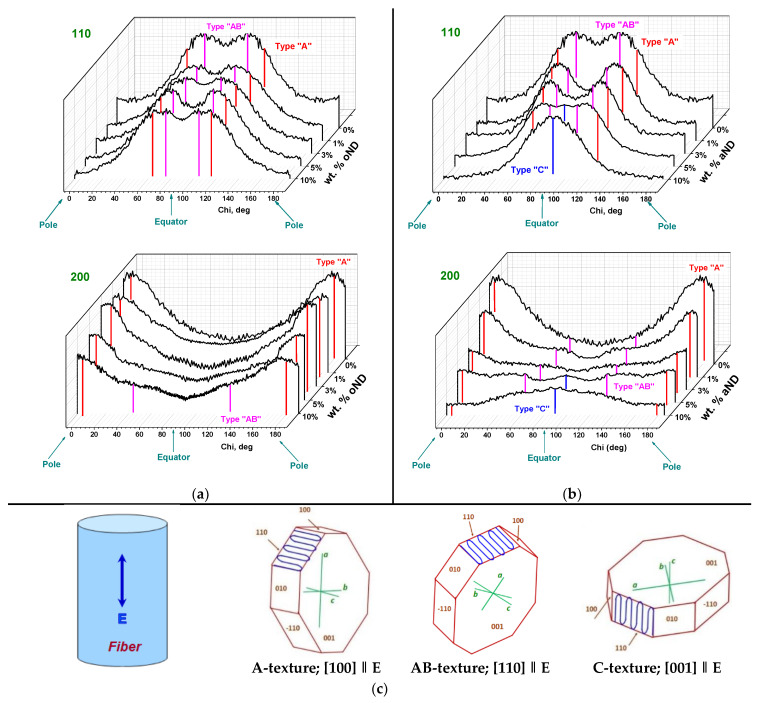
Azimuthal X-ray intensity distributions of the main reflexes, 110 and 200, recorded for the HDPE-based fibers: (**a**) HDPE + 5 wt.% oND; (**b**) HDPE + 5 wt.% aND; (**c**) schematic interpretation of the HDPE crystalline texture and macromolecular folding (blue line). **E** is the fiber extrusion direction.

Figure 10 shows the Chi-profiles of the azimuthal intensity distribution for the 110 and 200 maxima for all of the studied unoriented (DR = 1) HDPE-based nanocomposite fibers. Depending on the azimuthal location of these main peaks, one can draw a conclusion about the presence (or absence) of the HDPE crystalline texture (at the bottom of Figure 10, three possible types of the textures found in the investigated samples are sketched). The letters represent the crystallographic directions of the HDPE matrix crystalline parts that are preferentially aligned along the fiber extrusion direction (i.e., the fiber axis). If there are any displacements of the X-ray peaks along the azimuthal profiles recorded, it indicates the transformation of the texture from any type to the other one.

An analysis of the present azimuthal profiles of the HDPE composite fibers allows one to conclude that, in the fiber made of pure HDPE as well as that doped by the amorphous oND, two types of the texture (A or AB) are revealed. One should take note of the following very important point: in both cases, the orientations of the HDPE macromolecules folding and packaging are the most suitable for further unfolding during the high-temperature orientation drawing compared to those of the C-texture. However, when the number of the annealed nanodiscs (aND) in the composite fibers increases, the A-texture disappears completely, the AB-texture weakens strongly and the C-texture appears to become dominant in the HDPE-based fiber with 10 wt.% aND. Thus, one can conclude that the distinct change in the texture occurs in the composite fibers with the introduced aND nanoparticles. This interesting phenomenon is supposed to be ascribed to the structuring of the HDPE + aND melt, especially at high concentrations of the annealed nanodiscs. An effect of the melt-processing parameters on the structural transformations, especially the variations in the mechanisms of the crystallites orientations, was already described previously in a number of articles [17,40].

The authors of article [41] showed that the introduction of 2% SWCNTs dramatically changed the type of preferential orientation of the PE lamellae with the A-texture in pure PE to the C-texture in the composite fiber. Obviously, this is explained by the fact that SWCNTs were oriented along the fiber axis during the fiber production, while PE lamellae grew on them, forming shish-kebab type structures. In our work, it was possible to clearly show that, in order to transform the predominant orientation of the PE matrix crystallites, it is not necessary to incorporate longitudinal nanotubes there. The addition of the annealed carbon nanodiscs with nucleating ability can also lead to the transformation of the texture of the crystalline regions in the polymer. Apparently, this is associated with the change in the rheological properties of the composite melt (i.e., an increase in its viscosity) and, as a consequence, with the change in the stresses acting on the HDPE macromolecules in the flowing melt and in the mechanisms of polyethylene crystallization. As result, similar texture transformation was observed in the polyethylene fibers prepared from the melt with varied viscosity: the higher the HDPE + aND melt viscosity is, the more pronounced the C-texture is.

The detected transformation in the preferred orientation of the HDPE-matrix crystallites can affect the behavior of the composite fibers with the added aND nanofiller during the process of the high-temperature orientation drawing and, as result, the mechanical characteristics of the unoriented fibers. As noted earlier, such fibers with 10 wt.% aND could not be drawn up to DR = 8 because they mostly consist of the HDPE lamellae located in the very uncomfortable position for the further unfolding of the HDPE chains under an action of the orientating stretching.

In all of the oriented fibers (DR = 8), there is a re-crystallization and transformation of the lamellar structures in the fibrillar one. Therefore, as one would expect, there is one and the same type of texture, namely, the C-texture (i.e., all the crystallized parts of the HDPE macromolecules oriented along the fiber axis), so it does not make much sense to represent the Chi-scans of the oriented samples.

**Figure 11:**
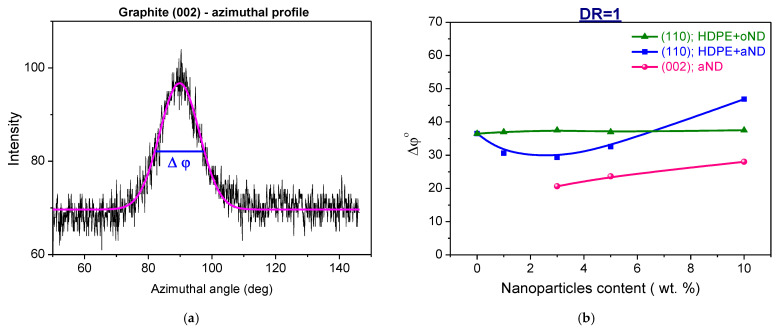
(**a**) An example of an azimuthal profile of the graphite 002 reflection of the aND nanoparticles (black line—experimental data; magenta line—computer fit line); (**b**) misorientation degree (Δϕ°) of the HDPE crystalline regions depending on the nanofiller content in the unoriented fibers (DR = 1).

Using the WAXS data presented in Figure 10 and similar data for the fibers drawn up to DR = 8, one can calculate the degree of crystallites misorientation (Δϕ°) with respect to the composite fiber axis. This value is the half-width of X-ray maximum along its azimuthal profile: generally, the higher the Δϕ°, the worse the orientation of the crystallites along the fiber axis. The dependence of the misorientation degree (Δϕ°) of the HDPE crystallites on the nanofillers content is shown in Figure 11.

As for the undrawn fibers (DR = 1), the misorientation degree of the main HDPE reflex (110) for the composite fibers filled with the original nanodiscs (oND) is independent of the number of nanoparticles in the matrix, whereas the Δϕ° for the samples doped by the annealed nanodiscs (aND) decreases first and then increases again (i.e., the HDPE lamella orientation is improved when the nanoparticles content is low, while at higher content, it becomes worse again). The misorientation angle of the peak corresponding to (002) reflections of aND tends to increase with an increase in their percentage—this fact can mean that the nanoparticles at higher contents may interfere with each other to be aligned along the fiber axis during the extrusion process, which will lead to the deterioration of the HDPE crystallites orientation.

Concerning the oriented fibers with DR = 8, the most important parameter is the degree of misorientation of the fibrils in the direction of the HDPE-based fiber, which can be estimated using HDPE reflex (002). It is found to have very low values of 4.5–5° for all of the investigated samples, almost independent of the nanofiller content. So, one can conclude that, in spite of the great differences in the initial HDPE-matrix lamellar structure, the orientation drawing process was quite effective, and there was a complete transformation of the lamella into the fibrils (this was also confirmed by SEM data earlier). Taking into account the SEM results, an important role is believed to be played by an extremely low adhesion between the nanofiller particles and the HDPE matrix, i.e., the nanoparticles themselves do not hinder the flow of the orientation processes of the macromolecules along the fiber axis.

As one might notice, there are some discrepancies in the results obtained: on the one hand, the undrawn composite fibers filled with the aND nanoparticles have the C-texture, which is expected to be the least favorable for further chain unfolding and transition to an oriented state; on the other hand, the oriented fibers filled with both oND and aND eventually have the same very good orientation of crystallites (the misorientation angle is very small and is only Δϕ° = 4.5–5°). We found an explanation for this very remarkable fact by studying these composite fibers with the help of the DSC method. These results will be published by us in the next paper (Part 2).

## 4. Conclusions

In the presented work, the HDPE-based fibers doped with the original and annealed carbon nanodiscs (oND and aND) were obtained by melt spinning followed by orientational drawing up to DR = 8. It was revealed that both types of the nanoparticles are well dispersed in the HDPE matrix and aligned along the fibers’ extrusion direction. Further, the carbon nanodiscs used demonstrate overly weak adhesion to the polymer matrix. Moreover, the annealed nanoparticles (aND) are found to possess a fine crystalline structure and can act as the nucleation agents that lead to the growing of the HDPE transcrystallites normally to their surface. 

It was observed that, in the pure HDPE fiber and in the composite fiber doped by the amorphous oND, two types of the texture (A or AB) are revealed by WAXS methods, while in the HDPE-based fiber with high amounts of aND, the C-texture becomes dominant. This fact allows one to conclude that not only the longitudinal carbon nanotubes/nanofibers but also the round nanoparticles are capable of transforming the predominant orientation of the morphological units in the melt extruded composite fibers. This is assumed to be associated with the change in the rheological properties of the composite melt (i.e., an increase in its viscosity) and, consequently, with the modification of the polymer crystallization conditions. It was found as well that the nanoparticles used do not hinder the flow of the orientation processes of the macromolecules along the fiber axis during high-temperature drawing.

Variation in the thermal and mechanical properties of the HDPE-based composite fibers with the incorporated carbon nanodiscs will be presented and discussed in further publications.

## Data Availability

Not applicable.
